# Behavioural responses of common dolphins to naval sonar

**DOI:** 10.1098/rsos.240650

**Published:** 2024-10-23

**Authors:** Brandon L. Southall, John W. Durban, John Calambokidis, Caroline Casey, James A. Fahlbusch, Holly Fearnbach, Kiirsten R. Flynn, Selene Fregosi, Ari S. Friedlaender, Samantha G. M. Leander, Fleur Visser

**Affiliations:** ^1^ Southall Environmental Associates, 9099 Soquel Drive, Suite 8, Aptos, CA 95003, USA; ^2^ Long Marine Laboratory, Institute of Marine Sciences, University of Santa Cruz, 115 McAllister Way, Santa Cruz, CA 95060, USA; ^3^ Hatfield Marine Science Center, Marine Mammal Institute, Oregon State University, 2030 SE Marine Science Drive, Newport, OR 97365, USA; ^4^ Cascadia Research Collective, 218 ½ W 4th Ave, Olympia, WA 98501, USA; ^5^ Hopkins Marine Station, Stanford University, 120 Ocean View Blvd, Pacific Grove, CA 93950, USA; ^6^ SR3 - SeaLife Response, Rehabilitation and Research, 2003 S. 216th St. #98811, Des Moines, WA 98198, USA; ^7^ Kelp Marine Research, Hoorn, CJ 1624, The Netherlands; ^8^ Royal Netherlands Institute for Sea Research, 1790 AB, Den Burg, The Netherlands

**Keywords:** noise, cetaceans, mid-frequency sonar, *Delphinus*, controlled exposure experiment

## Abstract

Despite strong interest in how noise affects marine mammals, little is known for the most abundant and commonly exposed taxa. Social delphinids occur in groups of hundreds of individuals that travel quickly, change behaviour ephemerally and are not amenable to conventional tagging methods, posing challenges in quantifying noise impacts. We integrated drone-based photogrammetry, strategically placed acoustic recorders and broad-scale visual observations to provide complementary measurements of different aspects of behaviour for short- and long-beaked common dolphins. We measured behavioural responses during controlled exposure experiments (CEEs) of military mid-frequency (3–4 kHz) active sonar (MFAS) using simulated and actual Navy sonar sources. We used latent-state Bayesian models to evaluate response probability and persistence in exposure and post-exposure phases. Changes in subgroup movement and aggregation parameters were commonly detected during different phases of MFAS CEEs but not control CEEs. Responses were more evident in short-beaked common dolphins (*n *= 14 CEEs), and a direct relationship between response probability and received level was observed. Long-beaked common dolphins (*n* = 20) showed less consistent responses, although contextual differences may have limited which movement responses could be detected. These are the first experimental behavioural response data for these abundant dolphins to directly inform impact assessments for military sonars.

## 1. Introduction

How noise from human activity can disturb and negatively impact marine life has been and remains a broad and significant scientific, conservation and international regulatory issue (see [1,2]). Some of the attention to this issue was generated by incidents coincident with active sonar systems used by navies around the world, specifically multiple lethal cetacean mass stranding events associated with military tactical mid-frequency active sonar (MFAS) [3–5]. The ‘mid-frequency’ range for these tactical sonars has conventionally been described as 1–10 kHz but the predominant energy for the SQS-53C sonars involved in most events occur within the approximately 3–4 kHz band. While direct hearing measurements are not available for all cetacean species, data from dozens of odontocetes suggest that, although this is outside the range of their most sensitive hearing, beaked whales, as well as the delphinid cetaceans evaluated in this study, are expected to have relatively good hearing in this band [1]. These strandings predominantly involved a few beaked whale species, particularly goose-beaked whales (*Ziphius cavirostris*). Beaked whales have consequently been the focus of subsequent experimental research into the cause of these strong adverse reactions (e.g. [6–9]). Although multiple events have been investigated, less evidence exists for lethal strandings coincident with MFAS in other marine mammal taxa (see [10]). However, MFAS exposure occurs regularly for many protected species, and sublethal responses that influence vital life functions may have meaningful consequences. Endangered baleen whales and deep-diving cetaceans including endangered sperm whales (see [11–15]) display clear sublethal behavioural responses in some conditions often related to changes in foraging.

Among cetaceans, delphinids include some of the most numerous, gregarious, behaviourally ephemeral and social species. Comparatively little is known about the behaviour of some of the most social oceanic delphinids in general because of the challenge of observing large, fluid and highly dynamic aggregations in the marine environment. Their small body size, hydrodynamic nature and capacity for bursts of speed (see [16]) mean that conventional, individual tag-based approaches to study detailed aspects of behaviour are difficult or infeasible. Additionally, individual-based methods may not represent the most appropriate approach given the highly social nature of these species. Group-based sampling methods may be more applicable given the context, like those employed in studies of schools of fish (e.g. [17]) or flocks of birds (e.g. [18]). Smaller bodied oceanic species such as short-beaked (*Delphinus delphis delphis*) and long-beaked common dolphins (*D. d. bairdii*) often occur in groups of mixed sex and age class, numbering hundreds or sometimes even thousands. Aspects of their population size and seasonal distribution have been relatively well-studied using broad-scale visual survey approaches in the Southern California Bight, our focal area (e.g. [19–21]). Both subspecies are extremely abundant, numbering in the hundreds of thousands (e.g. [22,23]).

There have been no direct measurements of the sensitivity to MFAS for any of the dozens of species of smaller delphinid cetaceans even though they collectively represent relatively large proportions of the total numbers of protected species predicted to be regularly exposed to Navy MFAS. There is no evidence that these impacts include the kinds of lethal strandings evident in other cetaceans or that sublethal effects for these populous species necessarily pose serious concerns for population consequences as has been raised for endangered species (see [13]). Responses in several delphinid species have been inferred from uncontrolled field observations of incidental exposures to operational MFAS [24] and controlled laboratory exposures of trained individuals [25]. These studies provide some confirmation that individuals of these species may respond to MFAS in some conditions. However, the lack of experimental control over potential response variables and the highly contextual nature of responses in captive contexts renders direct application of these results in management decisions difficult. Some level of responsiveness may be expected across social delphinids given how generally important acoustic communication is within their life history. But context and species response differences observed in other species (see [26]; [1,13,27]) and the highly social nature of many delphinids suggest that direct measurements within representative contexts are required rather than simple extrapolation from other species or contexts. This study provides the first such direct measurements within a species that is both commonly occurring and commonly exposed to naval MFAS.

To quantify aspects of the behaviour of large and ephemeral groupings of these species, we developed a novel integration of existing remote group sampling methods. These approaches were chosen to provide insight into different aspects of behaviour and enable us to begin to address some of the challenging issues related to studying fine-scale aspects of responses to disturbance in dynamic, prevalent, social delphinid species. This included an initial feasibility assessment demonstrating how group sampling methods could be synoptically applied to investigate group movement, acoustic behaviour and physiological responses to Navy MFAS on both fine and broad scales [28]. This approach integrated elements of previous controlled exposure experiment (CEE) approaches (focal follow, acoustics, spatial movement, behavioural state) and new elements of social behaviour, including group dynamics (speed, spacing, cohesion, subgroup configuration) on fine and broad scales. The integration of fine-scale spatial movement sampling builds on the increasing application of unoccupied aerial systems (UAS) in studying cetaceans (e.g. [29–31]).

This study applies and extends methods developed by Durban *et al*. [28] using approaches from earlier CEE studies and exposure methods developed to simulate some typical Navy MFAS systems to obtain a robust sample size of CEEs with an experimental source [32] and a smaller subset of exposures with actual operational systems (active tactical sonar systems dipped into the ocean from US Navy helicopters). Here, we investigate the response type and probability for two subspecies of common dolphins to these naval sonar signals exposure using fine- and broad-scale assessment of: (i) baseline variability in behaviour, (ii) the type, probability, magnitude and persistence of responses from realistic sonar exposures, and (iii) potential contextual differences within and between subspecies.

## 2. Methods

### 2.1. Overview

We used integrated remote visual and acoustic sensing approaches, applied at variable spatial scales [28], to quantify baseline behaviour and behavioural responses of dozens of groups of common dolphins (short-beaked (*Delphinus delphis delphis*) and long-beaked (*D. d. bairdii*) to simulated and operational MFAS. Subspecies included in each observation and experimental sequence were confirmed post hoc from high-resolution aerial images and photogrammetry measurements [33]. The integrated approaches included fine- and broad-scale visual and acoustic sampling methods to obtain a variety of synoptic and quantitative behavioural data in discrete experimental phases. Controlled exposure experiments consisted of sequential phases (‘pre-exposure’, ‘exposure’ and ‘post-exposure’) and included experimental sonar signals closely emulating tactical military sonars (see [1,15,32]), with similar power to operational naval sonar systems dipped from helicopters and no noise controls. To account for species’ dynamic yet synchronized gregariousness, groups and subgroups were the units of analysis rather than individuals. We evaluated the directionality and speed of focal groups, overall group acoustic behaviour and the configuration of subgroups within the larger group through phase-specific evaluations of behavioural state.

### 2.2. Field site

Experiments were conducted in offshore areas of the southern California Bight, primarily centred around Santa Catalina Island (subsequently referred to as ‘Catalina’). Field operations occurred during weeks-long field periods spanning June–December 2017–2021 (table 1). Most baseline and experimental sequences included vessel- and shore-based observations on and around Catalina, alternating locations in a phased manner to avoid sampling the same area on sequential days. Shore-based observations for broad-scale, full-group tracking, behavioural sampling and subgroup composition were conducted from several strategically selected platforms of known elevation. Most observations were collected from a primary base of operations at the University of Southern California’s Wrigley Institute for Environment and Sustainability. In cases where focal dolphin groups occurred outside the sampling range of shore-based observations, group-level data was recorded by vessel-based visual observers. UAS operations for fine-scale group sampling were based from vessels. Locations were selected based on focal group presence and acceptable sea state conditions, but CEEs were strategically rotated between possible sampling locations to reduce repeat exposures on consecutive field days.

**Table 1.** Controlled exposure experiments (CEEs) conducted for both short-beaked (top) and long-beaked (bottom) common dolphins. For each CEE, the total estimated group size (minimum-best-maximum estimates from shore-based visual observers) and unoccupied aerial system (UAS) focal (sub)group size are provided. Each CEE type, date/time and range (in km) from the source to the centre of the focal group (UAS location) is specified. Focal group modelled received levels (RL) in maximum root-mean-square (RMS) sound pressure level (SPL; dB re: 1 μPa) for any ping are provided for all MFAS CEEs, as well as cumulative sound exposure level (cSEL; dB re: 1 μPa^2^ s) across all pings. The availability of sufficient data (Yes (Y) or No (N)) of each categorical sampling type (UAS, passive acoustic monitoring (PAM), visual monitoring for subgroups) to conduct response analyses within each CEE is stated.

focal species	total (shore) group size (min-best-max)	focal (UAS) subgroup size	CEE no.	CEE type	CEE date and time (PDT)	range to focal (km) at CEE start	max RL (SPL)	aggregate RL (cSEL)	UAS data?	PAM data?	subgroup data?
short-beaked	50-80-200	200	2017_03	MFAS	10/1/17 (13:33:45)	3.0	141	151	Y	Y	N
short-beaked	150-200-225	75	2017_04	MFAS	10/2/17 (08:50:30)	2.9	137	152	Y	Y	Y
short-beaked	150-175-200	60	2017_06	control	10/4/17 (13:11:08)	1.6	n/a	n/a	Y	Y	Y
short-beaked	150-200-250	150	2017_07	MFAS	10/5/17 (09:05:07)	1.6	147	151	N	Y	Y
short-beaked	100-125-150	150	2017_08	control	10/5/17 (11:41:31)	1.5	n/a	n/a	Y	Y	Y
short-beaked	150-200-250	40	2019_02	control	10/23/19 (10:13:00)	0.6	n/a	n/a	Y	Y	Y
short-beaked	180-200-250	20	2019_05	MFAS (real Navy)	10/24/19 (13:48:59)	4.1	139	150	Y	Y	Y
short-beaked	180-200-250	300	2019_09	control	10/26/19 (12:15:00)	1.5	n/a	n/a	Y	Y	Y
short-beaked	250-250-300	25	2019_10	MFAS	10/27/19 (08:24:20)	1.3	149	165	Y	Y	Y
short-beaked	20-20-25	20	2019_11	MFAS (real Navy)	10/29/19 (11:32:00)	6.9	109	123	Y	Y	Y
short-beaked	300-400-500	150	2021_03	control	10/16/21 (09:32:20)	2.1	n/a	n/a	Y	Y	Y
short-beaked	150-200-220	250	2021_05	control	10/19/21 (14:56:00)	1.0	n/a	n/a	Y	Y	Y
short-beaked	150-150-175	150	2021_12	MFAS	12/6/21 (11:31:45)	1.6	152	168	Y	Y	Y
short-beaked	100-100-200	75	2021_13	MFAS	12/8/21 (10:14:34)	0.8	154	166	Y	Y	Y
long-beaked	700-900-900	12	2017_09	MFAS	10/06/17 (09:07:30)	0.8	153	165	Y	Y	Y
long-beaked	350-400-500	250	2018_03	MFAS	06/17/18 (07:14:40)	1.3	145	158	Y	Y	Y
long-beaked	300-400-500	200	2018_05	MFAS	06/19/18 (08:59:55)	2.7	153	165	Y	Y	Y
long-beaked	90-90-100	20	2018_08	MFAS	06/20/18 (14:03:41)	0.6	153	168	Y	Y	Y
long-beaked	12-12-15	6	2018_09	control	06/21/18	1.5	n/a	n/a	Y	Y	Y
long-beaked	250-300-350	20	2018_10	MFAS	06/21/18 (10:59:40)	1.7	148	163	Y	Y	Y
long-beaked	120-150-180	100	2019_01	MFAS	10/22/19 (11:04:03)	1.0	147	161	Y	Y	Y
long-beaked	100-200-250	200	2019_04	control	10/24/19 (09:45:50)	1.0	n/a	n/a	Y	Y	Y
long-beaked	70-90-110	12	2019_06	control	10/25/19 (09:10:15)	1.0	n/a	n/a	N	Y	Y
long-beaked	250-350-400	250	2019_07	MFAS	10/25/19 (11:13:46)	1.0	154	171	N	Y	Y
long-beaked	320-350-400	300	2019_08	MFAS	10/26/19 (09:25:00)	1.2	142	155	Y	Y	Y
long-beaked	n/a	150	2021_01	control	10/14/21 (11:48:02)	0.7	n/a	n/a	Y	Y	N
long-beaked	n/a	200	2021_02	control	10/15/21 (09:00:27)	0.5	n/a	n/a	Y	Y	N
long-beaked	200-300-300	150	2021_04	control	10/17/21 (09:20:40)	0.9	n/a	n/a	Y	Y	Y
long-beaked	350-400-400	200	2021_06	MFAS	10/20/21 (09:54:15)	0.6	141	151	Y	N	Y
long-beaked	150-250-250	120	2021_07	MFAS	10/21/21 (10:12:19)	1.8	149	162	Y	N	Y
long-beaked	200-250-250	30	2021_08	MFAS	10/22/21 (08:33:27	1.2	153	167	Y	Y	Y
long-beaked	20-40-40	20	2021_09	MFAS	12/3/21 (08:22:10)	1.1	157	172	Y	Y	Y
long-beaked	75-150-250	100	2021_10	MFAS	12/4/21 (09:26:22)	1.3	159	171	Y	Y	Y
long-beaked	n/a	19	2021_11	MFAS	12/5/21 (10:14:50)	0.7	153	169	Y	Y	N

### 2.3. Integrated remote sampling

We used complementary visual and acoustic sampling methods at variable spatial scales to measure different aspects of common dolphin behaviour in known and controlled MFAS exposure and non-exposure contexts. Three fundamentally different data collection systems were used to sample group behaviour (as in [28]). This integration allowed us to evaluate potential changes in movement, social cohesion and acoustic behaviour and their covariance associated with either the exposure to MFAS in known contexts and received level conditions or the absence of MFAS.

Broad-scale visual sampling of subgroup movement and configuration was conducted using theodolite tracking from shore-based stations. Assessments of whole-group and subgroup sizes, movement and behaviour were conducted at 2 min intervals from shore-based and vessel platforms using high-powered binoculars and standardized sampling regimes (as in [34,35]).

Aerial UAS-based photogrammetry quantified the movement of a single focal subgroup. The UAS consisted of a large (1.07 m diameter) custom-built octocopter drone launched and retrieved by hand from vessel platforms. For all the flights the median attitude was 60 m (range: 50–68). These altitudes were chosen to achieve a compromise of flying sufficiently low to provide highly detailed water-level pixel resolution (approx. 1.8 cm) but also high enough to cover a relatively large image footprint on the water to encompass all or most of the focal subgroup [28]. Behavioural reactions of dolphins to the drone at this altitude would be very unlikely, given results in these and other related species (e.g. [36,37]). However, this potential effect was controlled for as flights were made at similar altitudes during MFAS transmission (see below for description) experiments (median: 60 m; range: 50–68) and no-sonar control trials (median: 61 m; range: 57–67). The drone carried a vertically gimballed camera (at least 16 megapixels) and sensors that allowed precise geolocation of photographed dolphins, allowing spatially explicit photogrammetry to infer movement speed and directionality [28,29]. Photogrammetry images were collected every second, but speed and directionality parameters were modelled in 5 s blocks to match passive acoustic monitoring (PAM) data analysis (described below).

Three remote-deployed (drifting) PAM sensors were deployed in each experiment around focal groups from a small rigid-hulled inflatable boat with the goal of placing at least one recorder within 1 km of groups during each phase of the experiment while not influencing the behaviour of the dolphins. Identical methods for placement were used in sonar transmission and no-sonar control CEEs. Sensors were either SoundTrap ST300 (Ocean Instruments NZ, Auckland, New Zealand) sampled at 96 kHz or SNAP Recorders (Loggerhead Instruments, Sarasota, FL, USA) sampled at 44.1 kHz (see [38] for additional details on deployment and sampling). Five-minute WAV files were continuously recorded but binned into 5 s blocks for analysis (described below). These strategically deployed PAM sensors enabled us to examine both basic aspects of subspecies-specific common dolphin acoustic (whistling) behaviour [39] and potential group responses in whistling to MFAS on variable temporal scales [38].

### 2.4. Controlled exposure experiment methods

The overall methodology for CEEs was adapted from earlier studies with MFAS and tagged marine mammals (see [12]). This included a before-during-after (BDA) experimental design with a pre-exposure (confirmed no MFAS), exposure (MFAS, or control = no MFAS) and post-exposure (confirmed no MFAS) phase. MFAS and control CEEs were conducted in a randomized order within and between subspecies based on their occurrence, environmental conditions and the occurrence and location of previous MFAS transmissions from CEEs in this study. Adaptations of earlier experimental approaches for CEEs were made given both the unique context of group sampling for delphinids and the operational context (helicopter-dipping sonars) being simulated and, in several cases, tested. This primarily included shortening the MFAS (or control) exposure phases from 30 or 60 min each from earlier MFAS CEEs [1,7,14] to 10 min phases for this study, which was done for multiple reasons. First, it enabled consistent sampling across phases, given common dolphins' frequently rapid and variable group movement relative to sampling platforms. Second, the 30 min total CEE period across three phases could be encompassed within a single drone flight allowing fine-scale movement data to be obtained for the same subgroup(s) in all experimental periods. Third, the effective 10 min MFAS exposures during active transmissions closely replicated typical operational transmissions of helicopter-dipping MFAS operations, as communicated by Navy operational collaborators.

The experimental sound source used in projecting simulated MFAS signals was the same as that used in several earlier CEEs with tagged individual marine mammals [1,14,32]. Simulated MFAS signals were designed to mimic features of operational sonars based on direct input from the US Navy and consisted of three sequential continuous wave and frequency-modulated elements ranging from 3.5 to 4 kHz, with a total duration of 1.6 s and repeated every 25 s for the 10 min exposure period (up to 24 total pings). Like many operational MFAS tactical sonars, source levels were not ramped up but occurred at identical levels (212 dB re: 1μPa (root-mean-square (RMS); hereafter dB RMS) for all transmissions. Operational (AN/AQS-13) helicopter-dipping sonars differed slightly between sources in different CEEs, with similar frequency ranges (3–4.25 kHz) and had slightly higher source levels (unclassified source level = 215 dB RMS). These sources had very similar ping duration (1.5–2 s), harmonic structure, ping repetition (25–35 s) and total duration (10 min) during CEEs. Both simulated and operational MFAS sources were strategically positioned using real-time sound propagation analyses (see [1]) to achieve target received levels (RLs) for focal groups.

Given that these were the first CEEs with naval MFAS ever conducted on this species or any social delphinids, relatively low target RLs were tested. Previous regulatory and scientific assessments have proposed 160 dB RMS as a nominal RL at which a 50% response probability might be expected for ‘typical’ (non-sensitive) marine mammal species [13]. Using this value as a nominal upper bound for this study, target RLs for focal groups within larger aggregations were specified as 140–160 dB RMS. As identified in previous CEEs and in order to avoid creating spatial contexts that either reduced or enhanced the probability of response by either placing the source directly within or behind the track of moving animals [12,26,27], the objective was to achieve these target levels at the start of CEEs using *in situ* modelling approaches and placing sound sources perpendicular to the track of focal groups.

Received signals were measured at calibrated recorders at known locations and used to evaluate the efficacy of RL modelling approaches (see [28]) used to determine per-ping RMS values at the centre of focal groups, max dB RMS values during CEEs and per-ping and cumulative sound exposure level (SEL: dB re: 1 μPa^2^ s). Focal groups and any incidentally exposed marine mammals in the vicinity were visually monitored according to all specified permit requirements. If any marine mammals from focal or incidentally exposed groups came within 200 m of active MFAS sources, exposures were immediately terminated. This resulted in several CEEs having less than full transmission durations.

### 2.5. Analytical approaches

Three sets of quantitative response variables were analysed from the different data streams: directional persistence and variation in velocity of the focal subgroup from UAS photogrammetry; group vocal activity (whistle counts) from passive acoustic records; and number of subgroups within a larger group being tracked by the shore station overlook. We fit separate latent-state Bayesian models to each set of response data, with the models assumed to have two states: a baseline state and an enhanced state that was estimated in sequential 5 s blocks throughout each CEE [28]. For earlier years (2017–2018), whistle counts were only available in a randomized number of blocks and were otherwise treated as unknown. In 2019 and 2021, whistles were counted for all 5 s blocks (see [38]). The number of subgroups was recorded to evaluate overall group cohesion during periodic observations every 2 min and assumed constant across time blocks between observations. The number of subgroups was treated as missing data 30 s before each change was noted to introduce prior uncertainty about the precise timing of change. For movement, two parameters relating to directional persistence and variation in velocity were estimated by fitting a continuous time-correlated random walk model to spatially explicit photogrammetry data in the form of location tracks for focal individuals that were sequentially tracked throughout each CEE as a proxy for subgroup movement [28].

Movement parameters were assumed to be normally distributed. Whistle counts were treated as normally distributed but truncated as positive because negative count data is not possible. Subgroup counts were assumed to be Poisson distributed as they were distinct, small values. In all cases, the response variable mean was modelled as a function of the latent state with a log link*,*


$$log(Response_{t})=\lambda_{0}+\lambda_{1}Z_{t},$$

where at each 5 s time block *t,* the latent state took values of *Z_t_* = 0 to identify one state with a baseline response level *λ*_0_, or *Z_t_* = 1 to identify an ‘enhanced’ state, with *λ*_1_ representing the enhancement of the quantitative value of the response variable. A flat uniform (−30,30) prior distribution was used for *λ*_0_ in each response model, and a uniform (0,30) prior distribution was adopted for each *λ*_1_ to constrain enhancements to be positive. For whistle and subgroup counts, the enhanced state indicated increased vocal activity and more subgroups. A common indicator variable was estimated for the latent state for both the movement parameters, such that switching to the enhanced state described less directional persistence and more variation in velocity [28]. Speed was derived as a function of these two parameters [40] and was used here as a proxy for their joint responses, representing directional displacement over time.

To assess differences in the behaviour states between experimental phases, the block-specific latent states were modelled as a function of phase-specific probabilities, *Z_t_* ~ Bernoulli(*p*_phase[t]_), to learn about the probability *p*_phase_ of being in an enhanced state during each phase. For each pre-exposure, exposure and post-exposure phase, this probability was assigned a flat uniform (0,1) prior probability. The model was programmed in R (version 3.6.1; R Foundation for Statistical Computing), and the *nimble* package [41] was used to estimate posterior distributions of model parameters using Markov chain Monte Carlo (MCMC) sampling. Inference was based on 100 000 MCMC samples following a burn-in of 100 000, with chain convergence determined by visual inspection of three MCMC chains and corroborated by convergence diagnostics [42]. To compare behaviour across phases, we compared the posterior distribution of the *p*_phase_ parameters for each response variable, specifically by monitoring the MCMC output to assess the ‘probability of response’ as the proportion of iterations for which *p*_exposure_ was greater or less than *p*_pre-exposure_ and the ‘probability of persistence’ as the proportion of iterations for which *p*_post-exposure_ was greater or less than *p*_pre-exposure_. These probabilities of response and persistence thus estimated the extent of separation (non-overlap) between the distributions of pairs of *p*_phase_ parameters: if the two distributions of interest were identical, then *p* = 0.5, and if the two were non-overlapping, then *p* = 1. Similarly, we estimated the average values of the response variables in each phase by predicting phase-specific functions of the parameters


$$Mean.response_{phase}=exp(\lambda_{0}+\lambda_{1}p_{phase})$$

and simply derived average speed as the mean of the speed estimates for 5 s blocks in each phase.

To assess the evidence that the probability of movement response was related to the maximum MFAS received sound level (RL) across the *i* = 1,…,*n^MFAS^* CEEs with MFAS, but also accounting for potential relationships with the pre-exposure speed of the focal subgroup (v1), we fit a hierarchical regression model with RL and v1 as covariates,


$$\begin{array}{c} p(response\_move{)}_{i}=\alpha_{i}+\beta^{*}(RL_{i}–160)+\varepsilon_{i}\\ \alpha_{i}=\alpha^{0}+k^{*}(v1_{i}–mean(v1)) \end{array}.$$

Therefore, the probability of movement response was modelled as a linear function of RL, with the slope of the relationship given by *β*. The error distribution was assumed to be normally distributed within the truncated 0–1 parameter space because the response variable was a probability, with mean zero and a U(0,1) prior distribution on the standard deviation to allow for variability around the trend line. Because of centring the RL covariate values around a selected nominal value of 160 dB RMS (as discussed above, an RL value associated with some regulatory applications, including federal permits under which this study was conducted, to represent a reasonable 50% response probability threshold for untested, typical marine mammal species), the regression intercept represented the predicted probability of response based on the modelled data at an RL of 160 dB RMS. However, to account for the influence of pre-exposure speed, the intercepts *α_i_* were modelled as a linear function of v1, with slope given by *κ*, with the overall average *p*(response_move) at 160 dB RMS given by *α*^0^. We fit this hierarchical model using MCMC sampling implemented in the R package *nimble* and estimated the probability that the slope parameters (*β* and *κ*) departed from zero based on the proportion of MCMC iterations where the slope parameter was greater or less than zero.

## 3. Results

### 3.1. Field effort and controlled exposure experiments conducted

**Figure 1.**
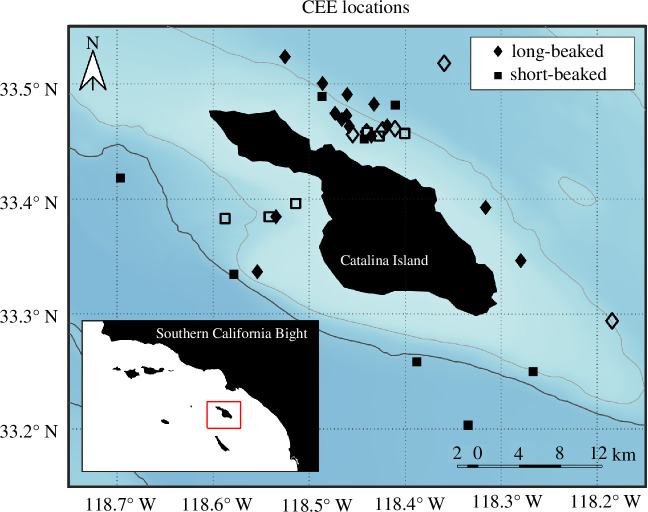
Locations of CEEs for long-beaked (diamonds) and short-beaked (squares) common dolphins in areas of the southern California Bight centred around Santa Catalina Island (‘Catalina’). Solid colour symbols indicate MFAS exposure CEEs. Open symbols indicate no-MFAS control CEEs.

Fieldwork was conducted primarily in autumn from October 2017 to December 2021 in areas throughout the Southern California Bight, centred around Catalina Island. Baseline visual monitoring and tracking of dozens of groups of both common dolphin subspecies were conducted ahead of CEEs to determine whether the configuration and behavioural state, tracking and other environmental factors (sea state, presence of other vessels) were within specified parameters for initiating CEEs. A total of *n* = 34 complete CEE sequences were conducted (22 MFAS and 12 control CEEs; table 1; figure 1).

As expected *a priori*, it proved somewhat challenging to achieve precisely consistent target levels across CEEs given the large group size and dynamic nature of these dolphins. However, the majority occurred within the overall target MFAS RL range (140–160 dB; table 1). We conducted *n* = 14 CEEs with short-beaked common dolphins (eight MFAS—six simulated and two operational Navy sources; six control CEEs) with source-focal group horizontal ranges at the start of CEE exposure phases of 0.6–3.0 km for simulated MFAS and control CEEs, and 4.1–6.9 km for Navy operational (helicopter-dipped) MFAS CEEs. With long-beaked common dolphins, we conducted *n* = 20 CEEs (14 MFAS—all simulated MFAS sources; 6 control CEEs) at starting source-focal group horizontal ranges from 0.6 to 3.0 km.

In some cases, logistical issues (equipment failure, groups moving beyond visual sampling range) precluded sufficient data acquisition from one sampling approach to be included in model assessments. However, given the integrated approach applied, this did not prevent model assessments of potential state changes in behavioural data from other data streams. Hundreds of hours of visual observational tracking and underwater passive acoustic data were obtained and processed during successfully conducted CEEs, along with nearly 44 000 individual aerial photogrammetry images from the drone (over 18 500 for short-beaked and over 25 000 for long-beaked common dolphins).

### 3.2. Group sizes sampled

Total sizes for groups sampled in CEEs were variable within and between subspecies, ranging from 12 to 500 individuals in short-beaked and 20 to 900 individuals for long-beaked common dolphins (table 1). Larger groups were not selected for CEEs, given the overall spread and logistical complexity identified in earlier pilot efforts, with some groups of more than 1000 animals. In many instances, the focal (UAS-followed) group represented a subgroup of the more extensive aggregation tracked by visual observers. Shore- and vessel-based visual observers provided minimum-best-maximum estimates of total group size for each CEE and an evaluation across all phases of the CEE of the number of distinct subgroups within each overall group.

### 3.3. Baseline (pre-exposure) behavioural parameters

For short-beaked common dolphins, modelled group speed (derived from velocity and directionality parameters, see [40]) during pre-exposure phases of MFAS CEEs (median: 10.5 km h^−1^; range: 7.7–17.1) was similar to pre-exposure speed for control CEEs (median: 9.7 km h^−1^; range: 8.1–20.7). Pre-exposure speed for long-beaked common dolphins was slightly higher and more variable for MFAS CEEs (median: 13.3 km h^−1^; range: 10.9–25.5) than for control CEEs (median: 12.7 km h^−1^; range: 10.1–14.6).

There was less variability in baseline (pre-exposure) values between CEE conditions for other response parameters. Median group whistle counts (see [38] for additional details) during pre-exposure conditions were generally low but had high overall variances across CEEs for both subspecies. For short-beaked common dolphins, group whistle counts within 5 s windows during pre-exposure phases for MFAS CEEs had a median of 0 (range: 0–98), while counts for control CEEs had a median of 10 (range: 0–22). For long-beaked common dolphins, whistle counts for MFAS CEEs had a median of 0 (range: 0–47), while counts for control CEEs had a median of 0 (range: 0–2). Subgroup counts (number of subgroups within the overall aggregation) from visual surveys during pre-exposure conditions were relatively similar for both species across CEEs. For short-beaked common dolphins, subgroup counts for MFAS CEEs had a median of 2 (range: 1–5), while subgroup counts for control CEEs had a median of 2 (range: 1–3). Long-beaked subgroup counts for MFAS CEEs had a median of 2 (range: 1–5), identical to values for control CEEs, which had a median of 2 (range: 1–5).

### 3.4. Exposure received levels for mid-frequency active sonar controlled exposure experiments

**Figure 2.**
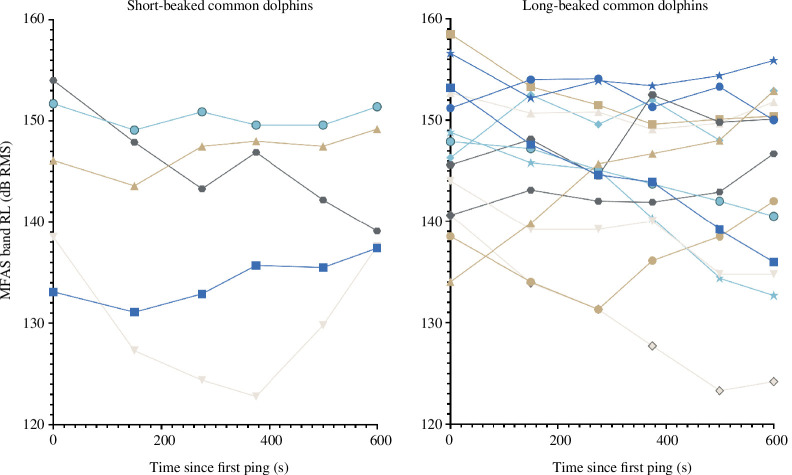
Common dolphin modelled received simulated MFAS levels during CEEs. Modelled exposure received levels (dB RMS) for sequential pings at approximately 2 min intervals during MFAS CEEs for focal groups of short-beaked (left panel) and long-beaked (right panel) common dolphins.

Modelled RLs for focal groups of each species in all MFAS CEEs are given in terms of maximum RLs (dB RMS) and aggregate (cSEL) values. For CEEs where complete MFAS transmission sequences were conducted, we provide a longitudinal time series of modelled (on-ping) RLs to illustrate overall patterns across exposures (figure 2).

Overall, focal groups were exposed to MFAS during exposure phases of CEEs within the specified 140–160 dB RMS target level below and up to the nominal *a priori* value associated with 50% response probability. Interestingly, while variable in occurrence and magnitude, for most MFAS CEEs, there is a negative (greater than −1 dB) slope in modelled focal group MFAS RLs from the initial ping to the sixth (next on-ping modelled RL) in the series (second data points in series shown here) for all (5/5) short-beaked common dolphin CEEs and most (9/14) long-beaked common dolphin MFAS CEEs. For control CEEs, no MFAS was present, but modelled ‘mock’ RLs presuming it was present suggest a different pattern over the first 2 min (six pings) of CEEs. Negative slopes in mock MFAS RLs for controls were observed in considerably fewer (1/6) short-beaked common dolphin and (2/6) long-beaked common dolphin control CEEs.

### 3.5. Modelled behavioural response variable results across experimental phases

#### 3.5.1. Short-beaked common dolphin results

**Figure 3.**
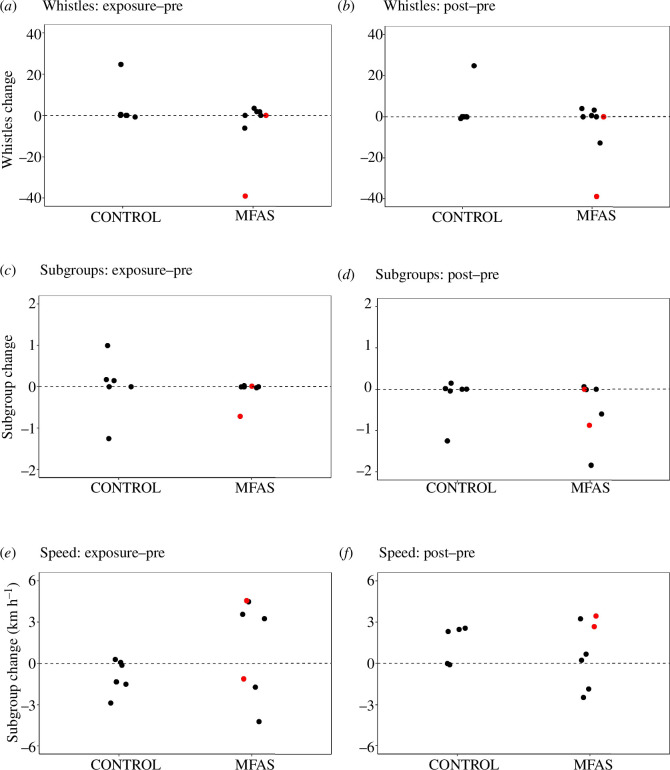
Short-beaked common dolphin behavioural response to MFAS exposure. Changes in the posterior mean estimate of modelled response variables between experimental phases for short-beaked common dolphins for control and MFAS CEEs. Differences in modelled group whistle, subgroup counts and group speed values are shown for exposure relative to pre-exposure phases (*a,c,e*) and post-exposure relative to pre-exposure phases (*b,d,f*). Red dots denote two MFAS CEEs conducted using operational Navy dipping sonar sources.

Changes in the posterior mean estimate of modelled response variables (whistle rates, subgroups, speed) for exposure and post-exposure phases relative to pre-exposure conditions for short-beaked common dolphins in control and MFAS CEEs are shown in figure 3.

Different response patterns for different behavioural parameters were observed for short-beaked common dolphins in terms of occurrence, probability and duration. These evident patterns are supported by model results, which confirmed the overall high whistle count variability, subgroup formation changes in a subset of the exposures and speed changes during MFAS (table 2). Modelled parameter values (with associated error) and changes in response values are shown in exposure versus pre-exposure and post-exposure versus pre-exposure values during control and MFAS CEEs for modelled group whistle counts, subgroups and derived group speeds. Conditions in which changes were detected (defined as instances where there is high degree of support (*p* > 0.9) of a difference in the phase-specific probabilities of being in an enhanced state) are indicated, and the number of changes detected as a proportion of CEEs in each condition is summarized.

**Table 2.** Short-beaked common dolphin response probability to MFAS. Behavioural metric summaries 2 for short-beaked common dolphins for modelled response variables (whistle counts, subgroup counts, UAS group speed) for pre-exposure, exposure and post-exposure phases. Estimates are presented as posterior means (posterior standard deviation). Bolded cells indicate CEEs for which there was a high degree of support (*p *> 0.9) of a difference in the phase-specific probabilities of being in an enhanced state. In some instances where total counts were relatively low overall, high probability changes occurred and are reflected with shading though differences were smaller than the number of significant digits reflected in the table.

CEE #	CEE type	group whistle counts (s.d.)			subgroup counts (s.d.)			group speed (km h^−1^) (s.d.)		
		pre-exposure	exposure	post-exposure	pre-exposure	exposure	post-exposure	pre-exposure	exposure	post-exposure
2017_03	MFAS	0.0 (0.1)	**1.9 (3.7)**	**4.0 (4.7)**	n/a	n/a	n/a	17.1 (14.7)	21.6 (14.6)	17.3 (13.8)
2017_04	MFAS	16.2 (2.6)	**19.6 (2.3)**	**19.4 (2.4)**	1.7 (0.1)	1.7 (0.1)	1.1 (0.1)	14.3 (9.7)	12.6 (11.5)	12.5 (11.3)
2017_07	MFAS	40.9 (2.6)	34.7 (6.6)	**28.2 (3.0)**	2.0 (0.1)	2.0 (0.1)	**2.0 (0.1)**	n/a	n/a	n/a
2019_05	MFAS^a^	47.0 (11.7)	**7.9 (5.2)**	**8.0 (5.4)**	4.9 (0.3)	**4.2 (0.2)**	**4.0 (0.2)**	10.2 (10.1)	**9.1 (5.2)**	12.9 (12.0)
2019_10	MFAS	0.0 (0.0)	0.0 (0.0)	0.0 (0.0)	1.3 (0.1)	1.3 (0.1)	1.3 (0.1)	15.5 (16.0)	**11.3 (12.4)**	13.1 (15.3)
2019_11	MFAS^aa^	0.0 (0.1)	**0.1 (0.2)**	0.0 (0.1)	1.9 (0.1)	1.9 (0.1)	1.9 (0.1)	6.7 (3.3)	**11.3 (9.2)**	**10.2 (7.3)**
2021_12	MFAS	0.0 (0.0)	**1.8 (3.2)**	**0.0 (0.0)**	1.3 (0.1)	1.3 (0.1)	1.4 (0.1)	10.5 (6.8)	**13.8 (10.0)**	11.2 (7.0)
2021_13	MFAS	0.0 (0.0)	0.0 (0.0)	**0.6 (0.5)**	3.7 (0.1)	3.7 (0.1)	1.9 (0.1)	9.4 (9.4)	**13.0 (13.4)**	**12.6 (12.2)**
2017_06	control	1.7 (2.1)	**26.4 (8.5)**	**26.5 (8.4)**	2.3 (0.1)	**1.0 (0.1)**	**1.0 (0.1)**	11.0 (9.9)	10.8 (11.1)	10.9 (10.4)
2017_08	control	0.1 (0.1)	0.0 (0.1)	**0.0 (0.0)**	1.0 (0.1)	1.0 (0.1)	1.0 (0.1)	8.3 (8.5)	8.6 (7.6)	10.6 (9.5)
2019_02	control	0.8 (0.9)	**0.1 (0.2)**	**0.0 (0.1)**	4.7 (0.2)	**5.7 (0.3)**	4.7 (0.2)	8.1 (3.4)	6.6 (3.6)	8.0 (6.7)
2019_09	control	0.0 (0.1)	**0.5 (0.7)**	**0.0 (0.0)**	1.0 (0.1)	1.0 (0.2)	1.0 (0.1)	20.7 (19.5)	17.8 (18.0)	**11.5 (13.9)**
2021_03	control	0.0 (0.0)	**0.0 (0.0)**	**0.0 (0.0)**	3.0 (0.2)	3.1 (0.1)	3.1 (0.2)	8.6 (4.5)	8.6 (4.0)	**11.0 (11.1)**
2021_05	control	0.0 (0.0)	**0.0 (0.0)**	**0.0 (0.0)**	1.1 (0.1)	1.3 (0.1)	1.1 (0.1)	13.6 (12.8)	12.3 (11.5)	16.2 (12.9)
no. changes/total MFAS CEEs		n/a	**5/8 (62.5%)**	**6/8 (75%)**	n/a	**1/7 (14.3%)**	**3/7 (42.9%)**	n/a	**5/8 (62.5%)**	**2/8 (25%)**
no. changes/total control CEEs		n/a	**5/6 (83.3%)**	**6/6 (100%)**	n/a	**2/6 (33.3%)**	**1/6 (16.7%)**	n/a	**0/6 (0%)**	**2/6 (33.3%)**

^a^ Denotes CEE conducted with operational US Navy dipping MFAS source.

For acoustic behaviour, changes in group whistle counts between phases were similar for control and MFAS sequences. However, there was an anomalous, extreme decrease in whistle rate during one of the operational naval MFAS CEEs, which occurred in the exposure phase and persisted into post-exposure. Given the high degree of variability observed in baseline patterns, as well as the highly ephemeral temporal nature of group whistling in common dolphins [38], using the entire (10 min) by-phase analysis window here probably limits the ability to detect finer-scale changes that may be occurring.

Relative subgroup changes suggest a delayed grouping response most evident during the post-exposure phases. While relative differences between exposure and pre-exposure phases are similar for control and MFAS CEEs, the number of subgroups was more commonly reduced post-exposure in MFAS (3/7) than in control (1/7) settings, showing the joining of two or more subgroups (figure 3*d*).

The most apparent evidence of short-beaked common dolphin behavioural changes during MFAS exposures exists for movement data sampled on focal UAS groups. Group speed during the exposure phase of MFAS CEEs became more variable than during control CEEs, with both strong increases and decreases detected (figure 3*e*). Changes are also evident in the post-exposure phase in both directions, although to a lesser extent than during the exposure phases.

#### 3.5.2. Long-beaked common dolphin results

**Figure 4.**
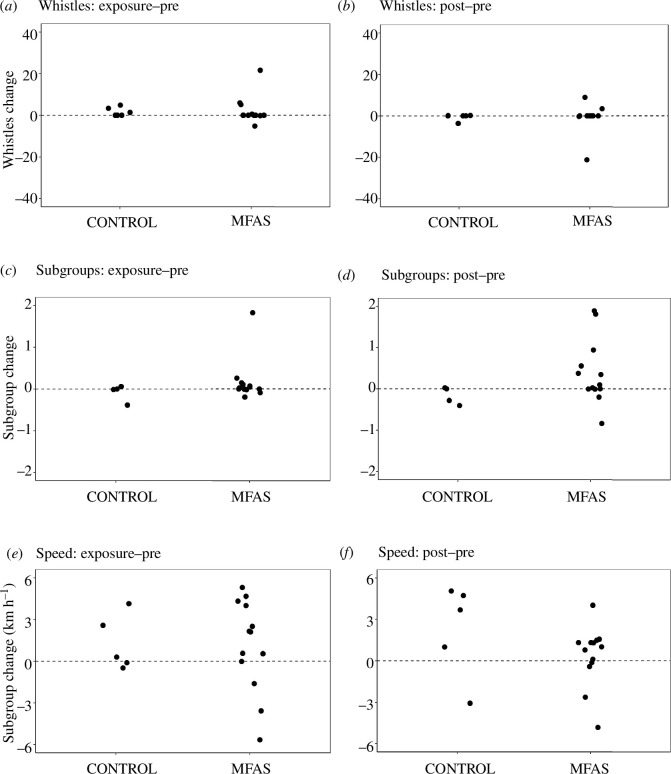
Short-beaked common dolphin behavioural response to simulated MFAS exposure. Changes in the posterior mean estimate of modelled response variables between experimental phases for long-beaked common dolphins for control and MFAS CEEs. Differences in modelled group whistle, subgroup counts and group speed values are shown for exposure relative to pre-exposure phases (*a,c,e*) and post-exposure relative to pre-exposure phases (*b,d,f*).

Changes in the posterior mean estimate of modelled response variables (whistle rates, subgroups, speed) for exposure and post-exposure phases relative to pre-exposure conditions for long-beaked common dolphins in control and MFAS CEEs are shown in figure 4.

Long-beaked common dolphins also displayed clear behavioural responses to MFAS to varying degrees relative to response variables type and exposure phase. These evident patterns are also supported by model results, which confirmed similar overall high whistle count variability, subgroup formation changes in some exposures (but in an opposite direction relative to short-beaked common dolphins) and some speed changes during MFAS (table 3).

**Table 3.** Long-beaked common dolphin response probability to MFAS. Behavioural metric summaries for long-beaked common dolphins for modelled response variables (whistle counts, subgroup counts, UAS group speed) for pre-exposure, exposure and post-exposure phases. Estimates are presented as posterior means (posterior standard deviation). Shaded and bolded cells indicate CEEs for which there was a high degree of support (*p *> 0.9) of a difference in the phase-specific probabilities of being in an enhanced state. In some instances where total counts were relatively low overall, high probability changes occurred and are reflected with shading though differences were smaller than the number of significant digits reflected in the table.

CEE no.	CEE type	group whistle counts (s.d.)			subgroup counts (s.d.)			group speed (km h^−1^) (s.d.)		
		pre-exposure	exposure	post-exposure	pre-exposure	exposure	post-exposure	pre-exposure	exposure	post-exposure
2017_09	MFAS	0.0 (0.0)	0.0 (0.0)	**0.0 (0.0)**	3.0 (0.1)	3.0 (0.1)	3.0 (0.1)	20.3 (13.8)	**14.6 (8.9)**	**15.4 (8.0)**
2018_03	MFAS	35.4 (3.6)	40.5 (2.8)	**14.2 (2.2)**	1.0 (0.1)	1.0 (0.1)	1.0 (0.1)	25.5 (30.8)	**29.5 (42.6)**	26.5 (36.8)
2018_05	MFAS	0.0 (0.0)	0.0 (0.0)	0.0 (0.0)	1.1 (0.1)	1.2 (0.1)	1.2 (0.1)	15.3 (17.7)	**17.7 (17.3)**	16.0 (18.8)
2018_08	MFAS	0.0 (0.0)	0.0 (0.1)	0.0 (0.0)	1.3 (0.1)	1.4 (0.2)	**2.2 (0.2)**	19.6 (14.7)	**16.0 (11.0)**	21.1 (13.7)
2018_10	MFAS	0.0 (0.0)	**6.0 (3.7)**	0.1 (0.1)	2.1 (0.2)	**3.9 (0.2)**	**3.9 (0.2)**	13.3 (9.5)	15.4 (14.2)	13.4 (10.6)
2019_01	MFAS	0.0 (0.0)	**0.5 (1.4)**	**3.4 (4.7)**	2.1 (0.1)	2.1 (0.1)	**2.5 (0.2)**	11.7 (8.5)	12.3 (9.6)	13.0 (10.3)
2019_07	MFAS	0.3 (0.4)	**0.0 (0.1)**	**0.0 (0.1)**	1.1 (0.1)	1.2 (0.1)	1.2 (0.1)	n/a	n/a	n/a
2019_08	MFAS	0.0 (0.0)	**0.0 (0.0)**	**0.0 (0.0)**	1.3 (0.1)	1.1 (0.1)	**1.1 (0.1)**	11.7 (12.0)	16.3 (12.9)	13.0 (12.3)
2021_06	MFAS	n/a	n/a	n/a	1.2 (0.1)	1.3 (0.1)	1.2 (0.1)	17.5 (12.1)	**22.8 (11.1)**	18.8 (11.1)
2021_07	MFAS	n/a	n/a	n/a	3.7 (0.2)	3.8 (0.2)	**5.6 (0.3)**	14.8 (9.8)	13.2 (8.6)	**12.2 (7.3)**
2021_08	MFAS	61.6 (1.6)	**83.3 (3.2)**	**70.7 (2.4)**	2.7 (0.2)	2.6 (0.2)	**3.0 (0.2)**	12.3 (3.9)	12.3 (4.1)	12.2 (3.9)
2021_09	MFAS	0.0 (0.0)	**0.0 (0.0)**	**0.0 (0.0)**	1.9 (0.1)	1.9 (0.1)	**1.1 (0.1)**	7.9 (3.5)	**12.2 (4.6)**	**9.4 (5.1)**
2021_10	MFAS	97.7 (4.5)	92.6 (7.2)	**8.4 (6.9)**	5.5 (0.2)	5.7 (0.3)	**6.0 (0.2)**	13.0 (11.6)	15.2 (10.2)	**17.1 (12.0)**
2021_11	MFAS	0.0 (0.0)	0.0 (0.0)	0.0 (0.0)	n/a	n/a	n/a	8.7 (3.7)	9.3 (3.0)	**8.3 (2.9)**
2018_09	control	0.0 (0.0)	0.0 (0.0)	0.0 (0.0)	1.6 (0.1)	**1.2 (0.1)**	**1.2 (0.1)**	11.3 (7.5)	**13.9 (4.4)**	**16.0 (6.4)**
2019_04	control	0.0 (0.0	**0.0 (0.0)**	**0.0 (0.0)**	1.0 (0.1)	1.0 (0.1)	1.0 (0.1)	10.0 (8.2)	10.3 (8.6)	11.0 (9.7)
2019_06	control	0.0 (0.0)	0.0 (0.0)	0.0 (0.0)	1.5 (0.1)	1.5 (0.1)	1.5 (0.1)	n/a	n/a	n/a
2021_01	control	0.1 (0.1)	**1.4 (1.4)**	**0.2 (0.3)**	n/a	n/a	n/a	13.8 (13.8)	**18.0 (12.9)**	**10.8 (7.6)**
2021_02	control	20.8 (1.1)	**24.1 (1.3)**	21.0 (1.2)	n/a	n/a	n/a	12.7 (11.7)	12.6 (12.6)	**17.8 (15.4)**
2021_04	control	22.3 (1.3)	**27.2 (1.6)**	**18.7 (1.1)**	3.4 (0.2)	3.3 (0.2)	3.1 (0.2)	14.6 (13.1)	14.1 (10.8)	**18.3 (20.4)**
no. changes/total MFAS CEEs		n/a	**6/12 (50%)**	**8/12 (75%)**	n/a	**1/13 (7.7)%**	**8/13 (61.5%)**	n/a	**6/13 (46.2%)**	**5/13 (38.5%)**
no. changes/total control CEEs		n/a	**4/6 (66.7%)**	**3/6 (50%)**	n/a	**1/4 (25%)**	**1/4 (25%**	n/a	**2/5 (40%)**	**4/5 (80%**

For acoustic behaviour, changes in group whistle counts between phases were similar for control and MFAS sequences. However, this also appears to be a function of the high variability observed in baseline patterns in the broad (10 min) by-phase analysis window [38].

Relative changes in long-beaked common dolphin subgroups suggest a delayed temporal response pattern, which becomes evident during post-exposure phases. Whereas slight variation was observed during exposure, more considerable differences in subgroup numbers became apparent in MFAS post-exposure (8/13 increased versus 1/4 during control). However, unlike the pattern for short-beaked common dolphins, more long-beaked common dolphin subgroups occur in MFAS post-exposure phases (i.e. groups split).

Long-beaked common dolphin group speed during the exposure phase of MFAS CEEs becomes much more variable than during control CEEs, with both substantial increases and decreases in modelled parameter outputs observed. However, similar patterns in CEEs with high probability of exposure phase parameter distributions differing from pre-exposure phases was observed for MFAS (6/13) versus control (2/5) CEEs. A similar pattern is detected during post-exposure phases.

### 3.6. Response contextual relationships: pre-exposure group speed and mid-frequency active sonar received level

Since some of the most apparent responses during MFAS exposures were evident in movement parameters, we evaluated some potential contextual variables that may be relevant in interpreting the drivers of these responses. We first evaluated and found no differences in response probabilities as a function of total group size, or UAS focal subgroup size, for either subspecies. Subsequently, we considered emergent relationships in the probability of detecting movement responses based on the pre-exposure speed of focal groups, the probability of such responses as a function of the maximum RL during MFAS exposures, as well as the potential interaction of these factors.

**Figure 5.**
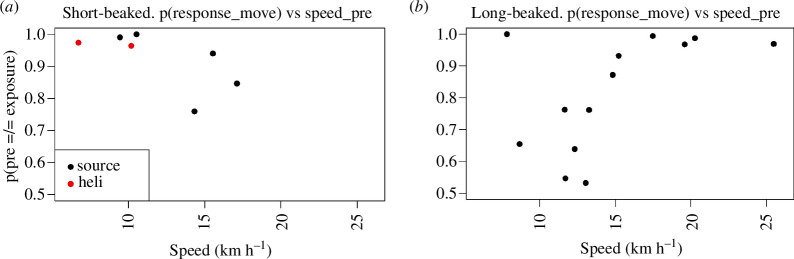
Probability of response as a function of speed. Probability of focal subgroups having different phase-specific probabilities of being in an enhanced state in the pre- and exposure phases (reflected by speed differing in exposure relative to pre-exposure phases) as a function of mean pre-exposure speed for short-beaked (*a*) and long-beaked (*b*) common dolphins.

We found direct relationships between the pre-exposure speed of focal groups and the probability of speed in the exposure phase being different (different phase-specific probabilities of being in an enhancement state) than during the pre-exposure (figure 5). Interestingly, these relationships occur in inverse patterns for short-beaked and long-beaked common dolphins, which may represent subspecies difference in baseline speed, but is more likely to be simply a function of the behavioural state conditions in which focal groups occurred when encountered and selected for CEEs. For short-beaked common dolphins (figure 5*a*), groups travelling slower in pre-exposure phases were more likely to change movement behaviour (typically by speeding up, but with an interesting example of further slowing in one of the two operational MFAS CEEs). Conversely, for long-beaked common dolphins (figure 5*b*), groups travelling faster in pre-exposure phases were more likely to change behaviour, typically by slowing down.

**Figure 6.**
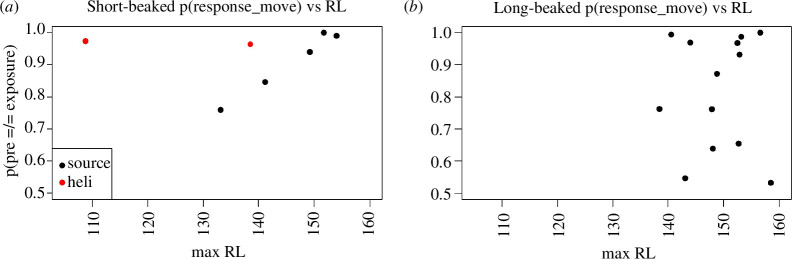
Probability of focal subgroups having different phase-specific probabilities of being in an enhanced state in the pre- and exposure phases (reflected by speed differing in exposure relative to pre-exposure phases) as a function of maximum modelled MFAS received level (RL; dB RMS) for short-beaked (*a*) and long-beaked (*b*) common dolphins.

We also evaluated the relationship of response probability for movement parameters during MFAS CEE exposure phases with the contextual exposure variable of exposure RL. Received levels (maximum values for any modelled MFAS ping; see figure 1) for short-beaked and long-beaked common dolphins relative to the probability of movement responses occurring during MFAS exposure phases are shown below (figure 6).

The probability of movement responses during MFAS exposure increased with RL for short-beaked common dolphins (except for the lowest RL dipping sonar CEE). However, it showed no clear relationship with RL for long-beaked common dolphins. Notably, however, many CEEs indicated a high probability of a movement response for both species at RLs well below the nominal 50% response probability at 160 dB RMS.

To evaluate this relationship further, given the strong relationships between pre-exposure speed and the probability of response for movement parameters, we evaluated the potential influences of this contextual factor with MFAS exposure RL. The regression model identified an increasing probability of movement response with increasing RL, most notably for short-beaked common dolphins (*p*[*β *> 0] = 0.80), with less evidence of a relationship for long-beaked common dolphins (*p*[*β *> 0] = 0.61). There was strong support for including pre-exposure speed in the model for the regression intercept underlying response probability, with *p*[*κ *< 0] = 0.94 for short-beaked common dolphins and lower response probabilities at higher pre-exposure speeds. There was an even more robust but inverse trend for long-beaked common dolphins with *p*[*κ *> 0] = 0.96, indicating higher response probabilities for focal groups with higher pre-exposure speeds. Without the inclusion of speed in a hierarchical model for the regression intercept, simple linear regression models of *p*(response_move) indicated little support for a relationship with RL (*p*[*β *> 0] = 0.44 and 0.52 for long and short-beaked common dolphins, respectively. Posterior estimates for overall regression intercepts *α*_0_ inferred that *p*(response_move) at an *a priori* estimated response 50th percentile threshold of 160 dB RMS was 0.94 (s.d. = 0.07) for short-beaked common dolphins compared with 0.83 (s.d. = 0.10) for long-beaked common dolphins. While the relationships between RL and speed relative to movement response probability are complex and differ between the subspecies, these estimates indicated a high probability of movement response for both at a nominal RL of 160 dB RMS.

## 4. Discussion

This study provides the first experimentally controlled measurements of behavioural responses of social delphinid species to military active sonars. By adapting newly developed experimental and analytical approaches [28], we identified behavioural responses of two subspecies of common dolphins during CEEs (*n* = 34) with MFAS exposures and no-noise controls. Our approach integrated complementary methods to sample different aspects of behaviour at different spatial and temporal scales. This included fine-scale measurements of the movement of individuals in high resolution, as well as group-level measurements of vocal behaviour and the overall cohesion of groups as indicated by differences in the number of subgroups. Differences in the ability to detect behavioural changes as a function of sonar exposure was highly temporally and context-dependent. Some behavioural parameters are highly variable over short time windows (e.g. group whistling) whereas others take longer periods to be expressed (e.g. group aggregation patterns). Within the 10 min phase windows evaluated quantitatively here, changes in subgroup movement and aggregation parameters were most readily observed and were commonly detected during different phases of MFAS CEEs compared with control CEEs in different patterns for each subspecies. Responses were more evident in short-beaked common dolphins, and a direct relationship between response probability and received level was observed. Long-beaked common dolphins showed less consistent movement responses, although contextual differences in pre-exposure speed may have limited the dolphin’s response options, or our ability to detect responses, but changes in group aggregations after MFAS exposure were clearly evident. These complex patterns clearly suggest that these globally abundant and often-exposed species probably respond to this sound source when exposed at comparable RLs within clearly variable natural behaviour patterns.

### 4.1. Delphinid response to mid-frequency active sonar

The most evident response for short-beaked common dolphin groups was a change in speed during MFAS exposure. Changes occurred in both directions, but given that many groups had low speeds during pre-exposure phases, there was a higher probability for slow-moving groups to change movement state by increasing speed. The probability of a change in group movement behaviour during exposure for short-beaked common dolphins was most directly related to the MFAS maximum RL for any of the response conditions for either subspecies. This was combined with a temporally delayed enhanced aggregation (fewer subgroups) in some groups, post-exposure. Acoustic responses were identified, but only on much finer (5 s) temporal scales evaluated separately [38]. Short-beaked common dolphin responses evident here were typically increases in speed and in some cases changes in cohesion and directionality in response to MFAS evident in visual observation and behavioural sampling (see spatial configurations for all CEEs; electronic supplementary material).

Long-beaked common dolphins showed a temporally delayed shift in group cohesion during MFAS, with groups splitting into more subgroups during post-exposure phases. Acoustic responses were identified subsequently as well, but only on much finer temporal windowed analyses [38]. Changes in movement were documented, specifically for faster-moving groups, which slowed down during exposure. The probability of detecting movement responses for long-beaked common dolphins was related to pre-exposure movement speed, with faster moving groups more likely to change states by slowing, an inverse pattern as observed for short-beaked common dolphins.

As expected for these fast, dynamic dolphins, baseline behaviour is highly variable, and behavioural changes occur at variable temporal scales. The integrated group sampling approach applied to evaluate spatial movement, group aggregation dynamics and acoustic behaviour revealed substantially different time courses, apparent subspecies differences and contextual dependencies. Despite these challenges, the combined results demonstrate differential responses between MFAS exposure and experimental control conditions, with differences in type and magnitude between subspecies and response variables. Changes in vocal behaviour occurring during most MFAS and control CEEs occurred for both subspecies in this study where the entire 10 min exposure periods was used as the analysis window. Differences between exposure and control conditions were therefore difficult to distinguish. Distinct responses to MFAS differentiated from control CEEs were, however, identified within finer (5 s) analysis windows [38]. Some of the most apparent contextual differences in responses between the subspecies relate to group movement and group aggregation conditions. Response types differed between subspecies in interesting and, in some cases, opposing ways.

It is notable that the overall CEE duration (30 min for all phases) and 10 min exposure phases are substantially shorter in this study than in most other MFAS CEEs with marine mammals (e.g. [1,7,12]). As described above, this shorter period was in part a function of the objective to fit the entire experiment within the duration of a single UAS flight, which is limited by battery duration. However, this was also done to emulate the particular type of operational Navy MFAS that was in two instances used in this study, namely AN/AQS-13 helicopter-dipping tactical active sonars. Source spectral and temporal parameters, contextual aspects including the stationary nature and lack of signal ‘ramp-up’, and the total duration of exposures were all very similar to (or were actually) these operational Navy MFAS systems. Given that our results indicate clear and consistent behavioural changes to both simulations of and actual MFAS signals from these dipping sonars, obvious next steps are the collection of additional data using helicopter-dipping sonars and simulations of and actual transmissions from the more powerful, longer duration MFAS exposures from Navy surface vessels (equipped with SQS-53C tactical MFAS). Such studies should arguably use longer sampling and exposure periods for consistency with earlier studies and the longer typical transmissions during training operations. Extending the duration of sampling using the methods developed by Durban *et al*. [28] and adapted and applied here could include either longer UAS flights with different or adapted platforms or the use of multiple flights or multiple drones.

Additional research in these and other related species is clearly needed to evaluate and amplify these initial results. However, responses identified in this first study with direct, experimentally controlled measurements strongly suggest that these social delphinids are more sensitive to noise disturbance than has been presumed from earlier, limited data. Specifically, we observed clear and, in some cases, strong and persistent behavioural changes to MFAS at much lower RLs, than previously presumed from observational or laboratory-based studies and currently predicted within exposure-response probability impact assessments for Navy sonar. In addition to informing additional studies, as described above, these new results should be considered and applied in exposure–responses functions now to better predict response probabilities for commonly exposed delphinid cetaceans.

### 4.2. Observations on controlled exposure experiments with large, dynamic groups

We faced the challenge of measuring fine-scale movement and acoustic behaviour in these dynamic species without archival or telemetered tag sensors, rather using an integrated group-sampling approach capable of evaluating whole group and subgroup parameters. Moreover, as for CEEs on other species, it can be challenging to control relevant contextual aspects of exposure. Although we sought to uniformly orient both monitoring sensors (acoustic recorders) and the experimental sound source at similar ranges and orientations to moving animals, their dynamic and unpredictable nature made this challenging to achieve in all CEEs (see spatial configurations for all CEEs; electronic supplementary material). Additionally, it was difficult to ensure CEEs were conducted in similar pre-exposure behavioural and environmental conditions within and between subspecies. The variability in pre-exposure group speed is an excellent example of this, where changes in speed may be more or less likely to detect simply as a function of the baseline state in which these CEEs were conducted. Other variable parameters identified that could similarly have context-dependent effects on response probability included group size and group demographics. We evaluated and found no differences in response probabilities as a function of total group size or UAS focal subgroup for either subspecies. However, given our limited experimental sample size and the challenges of characterizing subgroup size and dynamics within total group sizes in the many hundreds, these may be important mediating parameters that deserve further investigation and control in future CEEs. Further, in some CEEs, we observed environmental (e.g. groups encountering surface prey patches) and other (non-MFAS) anthropogenic factors (e.g. vessel movements, including those associated with the study) that may have temporarily influenced behaviour in portions of groups of these inquisitive animals. While we implemented specific protocols (e.g. avoiding CEEs during periods with other vessels present in close proximity to focal groups; limiting the speed and controlling proximity of our vessels as consistently as possible) to reduce the occurrence of these external perturbations and ensure consistency in MFAS and control CEEs, including terminating or excluding exposures, it is difficult to unequivocally conclude that such influences did not occur in some cases.

A key observation from this study is the variable time course over which baseline behaviour and responses may occur for different behavioural types. Acoustic (whistling) behaviour and responses were extremely ephemeral (seconds). Movement behaviour was dynamic on the temporal scale of the exposure (minutes). Changes in group cohesion (at least to the point where they were detectable) evolved most slowly (tens of minutes). Given the 10 min CEE experimental phases and by-phase analyses conducted here, it is unsurprising that the clearest responses detected were for movement parameters during MFAS exposures and subgroup changes in post-exposure phases. Additionally, to identify even potentially longer term (tens of minutes to hours) physiological impacts (e.g. blubber cortisol), tissue biopsy samples were collected at known periods following both MFAS and control CEEs, evaluated in a separate analysis.

### 4.3. Implications for management

Our results provide the first direct, quantitative exposure–response data for delphinids, with a robust sample size for marine mammal CEEs, and include exposure treatments of both simulated and operational Navy MFAS sources. These data have clear implications for impact assessments and management of military active sonar systems. Some caution and interpretation will be required in such applications given the initial nature of these studies, the challenges identified above, and the temporal and magnitude differences between response parameters for each subspecies. The relationships between MFAS exposure RL and response probability and magnitude are complex within and between subspecies for different behavioural parameters. However, the number of clear responses detected in many CEEs for MFAS well below the RL of 160 dB RMS previously considered to be required to result in a 50% response probability for ‘typical’ (non-sensitive) marine mammals is notable.

Substantial progress has now been made for these social delphinids in quantifying baseline behaviour and movement [38,39,43], physical and physiological parameters [33] and quantifying responses to disturbance ([28]; this study). However, given the number of delphinids that exist and the near total lack of information before these coordinated studies, there are many steps that need to be taken. Chiefly among these are expanding sample sizes and extending studies to other species. Pilot efforts have demonstrated the applicability of these approaches to Risso’s (*Grampus griseus*) and bottlenose dolphins (*Tursiops truncatus*), and a current effort is focusing on increasing sample size using similar methods for the later species. Also critically important is increasing sample sizes for operational naval sonars. We present two examples that serve as valuable demonstrations of the complex logistics required to coordinate tactical systems and dynamic species. However, additional data are needed, particularly given the low RLs associated with clear responses in many CEEs, notably the two we conducted involving operational Navy sonars. Continued integration of behavioural and physiological data (e.g. evaluating exposure RL relative to cortisol levels) is also an essential and ongoing consideration for assessing the health impacts from disturbance, which may have population consequences. Other key considerations for future studies include further analysis and potential control over CEEs for different group sizes and demographics, evaluation of responses to repeat exposures in known focal groups and more targeted efforts to conduct CEEs for groups in discrete behavioural states. Each question poses unique and complex challenges that will take years to address. Yet, this study achieved many objectives and provided direct empirical measurements on responses for taxa previously considered untestable in the field using conventional CEE methods. Until the current research programme, any such quantitative evaluation of behaviour and experimental evaluation of response for these abundant, dynamic, fast-moving delphinids was deemed unlikely or impossible.

## Data Availability

Data for each behavioural response parameter and associated code to replicate and run the latent-state models are available online [44].
